# Approaches to Characterize and Quantify Extracellular Vesicle Surface Conjugation Efficiency

**DOI:** 10.3390/life14040511

**Published:** 2024-04-15

**Authors:** Leora Goldbloom-Helzner, Harjn Bains, Aijun Wang

**Affiliations:** 1 Center for Surgical Bioengineering, Department of Surgery, School of Medicine, UC Davis Health, Sacramento, CA 95817, USA; legoldbloom@ucdavis.edu; 2 Department of Biomedical Engineering, UC Davis, Davis, CA 95616, USA; 3 Institute for Pediatric Regenerative Medicine, Shriners Hospitals for Children, Sacramento, CA 95817, USA

**Keywords:** targeted therapy, nanotherapeutic, extracellular vesicles, surface modification, conjugation efficiency, bulk analysis technologies, single-nanovesicle analysis technologies

## Abstract

Extracellular vesicles (EVs) are cell-secreted nanovesicles that play an important role in long-range cell–cell communication. Although EVs pose a promising alternative to cell-based therapy, targeted in vivo delivery still falls short. Many studies have explored the surface modification of EVs to enhance their targeting capabilities. However, to our knowledge, there are no standardized practices to confirm the successful surface modification of EVs or calculate the degree of conjugation on EV surfaces (conjugation efficiency). These pieces of information are essential in the reproducibility of targeted EV therapeutics and the determination of optimized conjugation conditions for EVs to see significant therapeutic effects in vitro and in vivo. This review will discuss the vast array of techniques adopted, technologies developed, and efficiency definitions made by studies that have calculated EV/nanoparticle surface conjugation efficiency and how differences between studies may contribute to differently reported conjugation efficiencies.

## 1. Introduction

Extracellular vesicles (EVs) are small membrane-bound nanovesicles that are released by nearly all cell types into their surrounding extracellular environment. There are three subtypes of EVs classified in accordance with their size and biogenesis pathway: exosomes, microvesicles, and apoptotic bodies. Exosomes are the smallest type of EVs, typically ranging in size from 30 to 150 nm. They are formed via the endocytic pathway, which involves the inward budding of the membrane of multivesicular bodies within the cell. Microvesicles, on the other hand, are larger than exosomes, with a size range of approximately 100 to 1000 nm. They are produced via the outward budding or shedding of the plasma membrane of cells. Apoptotic bodies are the largest type of EVs, measuring between 500 and 5000 nm. These vesicles are released from cells undergoing programmed cell death or apoptosis. All EVs are composed of a lipid bilayer membrane and contain a variety of bioactive molecules, including proteins, nucleic acids such as RNA and DNA, lipids, and metabolites [1,2]. EVs play essential roles in cell-to-cell communication, serving as carriers for the intercellular transfer of cargo molecules. They can be taken up by recipient cells and deliver their cargo, influencing the cellular behavior and physiology of the target cells. EVs have gained significant attention in recent years due to their involvement in various physiological and pathological processes [3,4]. They have been implicated in immune responses [5], angiogenesis [6], tissue regeneration [7], and cancer progression [8]. Furthermore, EVs have been found to be present in various body fluids, including blood, urine, and saliva, making them attractive targets for non-invasive diagnostic and therapeutic applications [9]. Although EVs show promise as targeted drug delivery systems, a key challenge lies in their biodistribution in vivo, with ineffective targeting and suboptimal accumulation at disease sites often limiting their therapeutic potential [10,11,12,13,14,15]. Modifying the specificity of EVs or synthetic nanoparticles to recipient cells is a key strategy being explored, and approaches to optimize their delivery are currently under investigation [16,17,18,19].

Many studies have explored the surface modification of EVs to enhance their targeting capabilities [4,20,21,22,23]. Most studies report physical and biomarker characterization of EVs or nanoparticles post-conjugation to explore how conjugations affect size, zeta potential, morphology, and function (uptake or therapeutic potential) [24,25,26,27,28,29,30]. However, we found few studies in the literature that seek to confirm how efficiently their nanoparticle sample is conjugated with a targeting molecule of interest [31,32,33,34,35,36,37]. Additionally, to our knowledge, there are no well-established standard practices or assays shared by all to make this calculation. To confirm the successful surface modification of targeting molecules onto extracellular vesicles, researchers have utilized numerous techniques and approaches. In addition to different techniques and approaches, various calculations have been used to represent nanoparticle surface conjugation efficiency. Some use the percentage of total EVs labeled with at least one targeting molecule, while others report the average number of targeting molecules conjugated onto a single nanoparticle.

This review will comprise an overview of the vast array of techniques used, technologies developed, and efficiency definitions made by studies that have calculated EV/nanoparticle surface conjugation efficiency (Table 1). To our knowledge, this is the first study to compare nanoparticle conjugation findings and efficiency calculation techniques used in the literature.

## 2. Conjugation Methods

Researchers have found numerous ways to modify the surface of EVs/EV mimics for improved tracking and therapeutic targeting purposes. Surface conjugation has taken on forms including physical, chemical, and biological modification [20].

Physical modification techniques involve altering the EV surface via processes such as extrusion, sonication, or electroporation to fuse or create engineered nanoparticles. Extrusion, where the vesicles are passed through small pores or filters to alter their size, shape, and surface characteristics, has been used to create EV–mimetic nanovesicles from cells modified with molecules of interest [26]. In other instances, extrusion has been used to fuse liposomes and EVs together to form hybrid EVs with engineered surface molecules and loaded cargo [29]. Sonication and electroporation are commonly used to load cargo into EVs [30,43,51].

Chemical conjugation methods involve attaching specific molecules or functional groups to the surface of EVs using chemical reactions. These methods enable the precise and controlled modification of vesicle surfaces. Click chemistry, or azide alkyne cycloaddition, is one such approach where an alkyne moiety reacts with an azide group to form a stable triazole linkage. This is an efficient bioorthogonal reaction that is utilized to selectively and covalently link molecules to EVs [28,31,32,42,44,45,49,52]. Another chemical conjugation method is amine crosslinking, which involves the reaction between amine groups on the vesicle surface and functional groups on the molecule to be conjugated [28,33,34,44,45,46,49,50]. Similar to amine crosslinking is maleimide-thiol reactions. This method involves the reaction between maleimide groups on the EV surface and thiol groups on the molecule to be attached [32,36,37]. These bioconjugations are specific and require only mild conditions that do not damage EV integrity. Lastly, lipid insertion techniques involve incorporating desired molecules into the lipid bilayer of the EVs—usually under higher temperature (40 °C) incubation conditions [33,35,36,41,46]. This method is sometimes preferred as it is nonselective in its conjugation versus other chemical conjugation techniques, which require the availability of certain chemical groups on EV surfaces for proper and efficient surface modification.

Biological surface modification includes harnessing receptor interactions to modify EV surfaces and indirect labeling via cell transfection/downstream isolation of transfected/engineered EVs. Some target molecules have been added to EV surfaces via the use of CD63 binding—a tetraspanin biomarker normally expressed on EVs [24]. Other methods have transfected cell membranes with plasmids to increase expression of certain target molecules that are linked to components found on EVs, such as Lamp2b and lipid raft enriched glycosylphosphatidylinositol (GPI)-anchored proteins [25,29,38,39,48].

These advancements in modifying EVs have opened up new possibilities for targeted drug delivery. Within each conjugation efficiency analysis technique discussed in this review, the conjugation method will be referenced to highlight the potential factors that could lead to different results in conjugation efficiency.

## 3. Bulk Analysis Technologies

**Figure 1:**
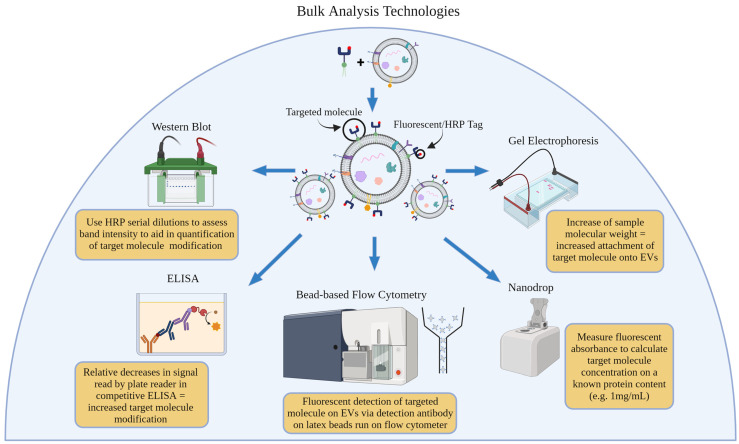
Overview of bulk analysis technologies used to quantify EV conjugation efficiency and the fundamental principle behind each technique’s calculation.

Bulk analysis technologies refer to techniques that allow for the analysis of a bulk sample. In the context of using extracellular vesicles, bulk analysis technologies can analyze a large number of extracellular vesicles in a sample at once, providing information about their size, composition, and other characteristics [53]. These techniques can provide a yes or no answer to the question “are at least some EVs in this sample conjugated with the molecule of interest?” (Figure 1).

### 3.1. Western Blot

Western blotting has been used to detect specific proteins in biological samples and has begun to be used to confirm the presence of surface-conjugated molecules on nanoparticles [54]. Targeting molecules have been modified with recognizable molecules that aid in the isolation and identification of modified EVs. One such study used Western blot analysis to confirm surface modification of dendritic cell (DC)-derived EVs with a FLAG epitope encoded in a plasmid to be expressed on exosomal marker Lamp2b. Protein-A Sepharose beads were coated with anti-FLAG antibodies, and engineered EVs were incubated with these beads as part of a pulldown assay. Researchers showed that untransfected DC-derived EVs, although positive for Lamp2b expression (based on results from a Lamp2b-coated pulldown assay), were negative for FLAG expression, which supported the claim that EV modification was successful [38].

Other recognizable protein tags have been modified on targeting molecules for ease of antibody use in Western blot stains. Hemagglutinin (HA) molecules have been transfected within plasmids into cells. Then, secreted EVs were run on Western blots using anti-HA antibodies to confirm the presence of the targeting molecule of interest. Ohno et al. used pDisplay vectors to transfect human embryonic kidney cells (HEK293) with HA-expressing GE11 or epidermal growth factor (EGF) peptides fused to the cell’s transmembrane domain of platelet-derived growth factor receptor. The cell-secreted EVs were isolated and analyzed for expression of HA molecules using Western blot analysis. Their results revealed bands of predicted size on engineered EVs compared to a lack of bands for empty vector-engineered EVs and unmodified EVs [39]. Kooijmans et al. also used HA-aided Western blot analysis to confirm the presence of N-terminal HA-tagged anti-EGFR nanobodies on EVs secreted from transfected Neuro2A cells. These nanobodies were modified with an N-terminal HA-tag, which allowed the researchers to see the presence of the nanobodies on EVs via Western blots that used anti-HA antibodies [25]. This group also began using c-Myc tags to identify the presence of EGa1 nanobodies on PEG micelles. First, the researchers used Western blots to demonstrate the increase in molecular weight of an Myc-tagged nanobody band, indicating the successful chemical conjugation of nanobodies to PEG–phospholipids of the micelle. Then, the researchers confirmed the incorporation of nanobody–PEG–lipids into EVs via lipid insertion, followed by size exclusion chromatography (SEC) to purify EVs. By increasing the lipid insertion incubation temperature from 4 °C to 60 °C, the researchers showed increasingly dark Western blot bands aiding in the optimization of their EV conjugation protocol [43]. Further optimization of conjugation methods was taken when this group used Western blots to show that amounts of Myc-tagged EGa1-C1C2 nanobodies coeluted with EVs post-SEC in a concentration-dependent manner [40].

Although some studies have modified targeting molecules with protein tags, some studies use commercially available primary antibodies against the exact targeting molecule of interest. In a study that created hybrid EVs to present recombinant programmed cell death 1 (PD-1) protein and baculoviral fusogenic glycoprotein gp64, researchers used anti-PD-1 (ab89828, Abcam, Cambridge, UK) and anti-gp64 (sc-65499, Santa Cruz Biotechnology, Santa Cruz, CA, USA) antibodies in their Western blot stains. Their results confirmed the presence of both proteins on hybrid EVs post-fusion with cargo-loaded liposomes [29].

#### Western Blot (Serial Dilution of HRP)

Western blot analysis is generally a bulk analysis technique and does not naturally produce a quantitative output. Although the bands provide a mostly qualitative analysis of a sample’s protein content, studies have explored the use of HRP serial dilutions to aid in the quantification of peptide modification on EV surfaces. In one study, researchers determined the amount of biotinylated epidermal growth factor receptor (EGFR) targeting peptide, B-TL5, attached to red blood cell-derived EVS (RBCEVs) using HRP-conjugated streptavidin in a Western blot. The researchers compared the biotin signals from conjugated RBCEVs to a dilution series of dibiotinylated HRP. Using this comparison of bands, researchers were able to report that an average of approximately 380 copies of peptides were ligated to each RBCEV [30]. The authors made it clear that the number of conjugated peptides per EV was an average and not an exact number of peptides conjugated to every RBCEV. Western blot is unable to identify the percentage of EVs that were labeled and is limited by its assumption that every EV is conjugated at the same rate. This research group continued to assess the conjugation of a new peptide, B-T140, which targets common cancer marker CXC-chemokine receptor 4, using the same HRP standard curve established previously. This study also conducted optimization strategies, identifying parameters in the ligation reaction such as pH, temperature, and peptide concentration that changed the ligation efficiency considerably. The optimized conjugation was reported to be an average of over 1000 B-T140 peptides per EV [27].

### 3.2. Bead-Based Flow Cytometry

Bead-based flow cytometry was developed to measure the presence of fluorescent antibodies on nanoparticles without the need for flow cytometers to be calibrated on a nanoscale [55,56]. Beads of varied material and of diameters on the µm scale are coated such that incubation with nanoparticles—especially EVs—are immobilized onto the beads’ surfaces. This technique is regularly used for EV characterization for biomarker expression. In recent years, the bead-based flow has been another bulk analysis technology to be employed for the confirmation of successful nanoparticle conjugation [24,42,47,50]. In the study, conjugating GE11 or EGF peptide onto HEK293 EV, EVs were incubated with latex beads, and Myc-tagged peptides were counted using Alexa Fluor-488 conjugated anti-Myc antibodies. Antibodies against CD81 were used as a positive control for EVs. Samples run on the FACSCalibur system showed that between 65 and 75% of the beads were positive for Myc tags and were therefore the peptides of interest [39]. Similarly, FACS analysis of RBC EVs conjugated with biotinylated EGFR-targeting peptides resulted in 77.2% EV-bound latex beads positive for Alexa Fluor 647-conjugated streptavidin [30].

Although bead-based flow cytometry cannot identify the exact conjugation efficiency of molecules on EVs on a single nanoparticle level, studies have confirmed the stability of conjugation over time and have shown that varied conjugation parameters can improve percentages of fluorescently tagged beads—indicating higher EV conjugation efficiencies [47]. In a study modifying EV surfaces with ssDNA tethers, the authors used CD63-conjugated magnetic beads for the immobilization of EVs. Cy5-labeled DNA conjugated to cholesterol molecules (for lipid insertion) was varied between 0 and 20 μM, and corresponding fluorescence intensities, as read by the flow cytometer, increased, indicating an improvement in EV-Chol-ssDNA conjugation efficiency. Calculations made by the authors estimated that DNA tether–conjugation onto EV surfaces increased from 1800 to 6900 tethers per EV as ssDNA concentration increased. Once concentrations increased above 20 μM, the authors observed aggregation and micelle formation, leading to reduced DNA tether insertion. Bead-based flow cytometry was also used to confirm the stability over time of ssDNA tethers on EV surfaces at 4 °C and 37 °C [35].

### 3.3. Absorbance

Several techniques have been employed to determine the absorbance of conjugated molecules along the UV–visible spectrum on EV/nanoparticle samples for which the protein content/concentration was known. These values, together, allow authors to estimate a concentration or average absolute number of molecules modified on nanoparticles.

#### 3.3.1. Nanodrop/BCA

In a study that conjugated azide-presenting EVs with DBCO-Cy3 via click chemistry methods, Cy3 conjugation efficiency was calculated by measuring the fluorescent absorbance of Cy3 using Nanodrop on a known protein concentration of 1 mg/mL EVs (as determined by BCA assay). Normalized to this 1 mg/mL EV protein content, the Cy3 concentration was calculated to be 790 nM and 440 nM for the two azide molecules (AHA and ManNAz) incorporated into EVs [42]. Using HPLC/UV visible absorbance, another study identified ~1.5 alkynes per 150 kDa protein that were available to react to an incubated azide–fluor 545 molecule. They estimated this using liposomes modified with varying concentrations of terminal alkynes to extrapolate into a standard curve [49]. Another study used a BCA standard curve to calculate the efficiency of a click reaction of Alexa Fluor-488-modified monoclonal antibody fragment TRAZ–azide onto alkyne-presenting PLGA–PEG nanoparticles (NPs). The authors showed that this modified TRAZ Fab-NP had a conjugation efficiency of 193.1 TRAZ F(ab) pmoles/mg PLGA-PEG NPs or 18.4% modified TRAZ F(ab) [31].

#### 3.3.2. Fluorescence Correlation Spectroscopy/Plate Reader/ELISA

Fluorescence correlation spectroscopy (FCS) analysis has also informed optimization of nanoparticle conjugation. To improve Fabs conjugation onto polyion complex (PIC) micelles, one study varied azide groups present on micelles using maleimide-thiol chemistry and incubated their samples with DBCO-Alexa Fluor 647 molecules to establish an average number of azide groups available for conjugation per micelle. Then, antigen-binding fragments (Fabs) were conjugated onto PIC micelles, followed by incubation with DBCO-Alexa Fluor 647 to label any unreacted azide groups. The reduction in fluorescent absorbance in the 647 nm spectra represented the conjugation efficiency of Fab onto PIC micelles. Although DBCO-Alexa Fluor 647 molecule incubation alone demonstrated that ~ 10 azide groups were present on PIC micelles, optimized protocols could only reach at most 1.5–3.5 Fabs per PIC micelle. The authors also used UV-vis absorbance to determine conjugated Fab content in micelles. Protein (A280) and polymer (A555) absorbance were measured, and similar ranges of 1–3 Fab molecules per micelle were confirmed. Authors suggested that steric hindrance from larger Fabs could contribute to the 20–30% conjugation (normalized from dye molecules) [32,57]. Plate readers have also been used to read fluorescent values for known EV samples to determine standard curves for conjugation analysis. One study conjugated EVs with Rhodamine-labeled CatK Binding Peptide (CKBP)-azide sequences. Based on the standard curve they determined, the authors reported that 0.22 μg CKBP was present per 10μg of EV [45]. Another study estimated the number of fluorescently tagged αvβ3-targeting peptides c(RGDyK) on modified bone marrow-derived mesenchymal stromal cell EVs by comparison to a fluorescent standard curve of free c[RK(FITC)DyK]. It was calculated that 1 mg/mL modified BMSC EVs contained an average of 523 nM peptides [44]. Relative decreases in signal in a competitive ELISA also aided one group in the calculation of peptide copy number in a known EV sample. EVs conjugated with biotinylated peptide (B-TL5 or B-T140) were immobilized on streptavidin-coated plates. The plate was then incubated with biotinylated HRP. A standard curve was determined by serial dilutions of free biotinylated peptides, and the authors reported ~351 B-TL5 peptides per EV and ~1402 B-T140 peptides per EV [27].

#### 3.3.3. Gel Electrophoresis

Gel electrophoresis has also been used to confirm conjugation on EVs. Especially in the case of using DNA/RNA aptamers, this technique is especially useful to show the presence or lack of bands post-nanoparticle conjugation. A study ran an MUC1 aptamer-decorated EV on an agarose gel. When the aptamer was conjugated to EV samples, the authors observed that the molecular weight of the fluorescent band increased as compared to the free aptamer, which had a lower molecular weight and therefore traveled farther down the gel. Based on a Nanodrop assay, the authors were able to determine that on 100 μg of EV protein, 44.78 μg MUC1 aptamer was present in the sample [34]. A similar study conjugated mannose onto bovine serum EVs but substituted biotin for mannose for calculated conjugation efficiency. NHS-PEG-biotin was reacted with EV surface protein amine groups, or DSPE–PEG–biotin was inserted into the EV’s lipid membrane, and streptavidin-FITC (stv-FITC) was incubated with the EV samples. When run on 10% polyacrylamide gel, free stv-FITC produced a single strong band at 55 kDa, whereas EXO-PEG-bio samples incubated with stv-FITC produced two bands—a faint band at 55 kDa and a stronger band shifted higher on the gel indicating successful conjugation with the larger nanoparticles. Based on analysis of each band’s fluorescence, the authors were able to estimate a 40% and 70% EV conjugation using NHS-PEG-bio or DSPE-PEG-bio, respectively. The authors also used a biotin quantification kit to quantify available biotin binding sites on EV samples. A Bradford assay kit quantified EV protein content, and the authors were able to determine that, depending on incubation ratios of EV to biotinylated linker molecule, there was 12–250 nmol DSPE-PEG-biotin/mg EV protein and 40–400 nmol NHS-PEG-biotin/mg EV protein [33].

## 4. Single-Nanovesicle Analysis Technologies

**Figure 2:**
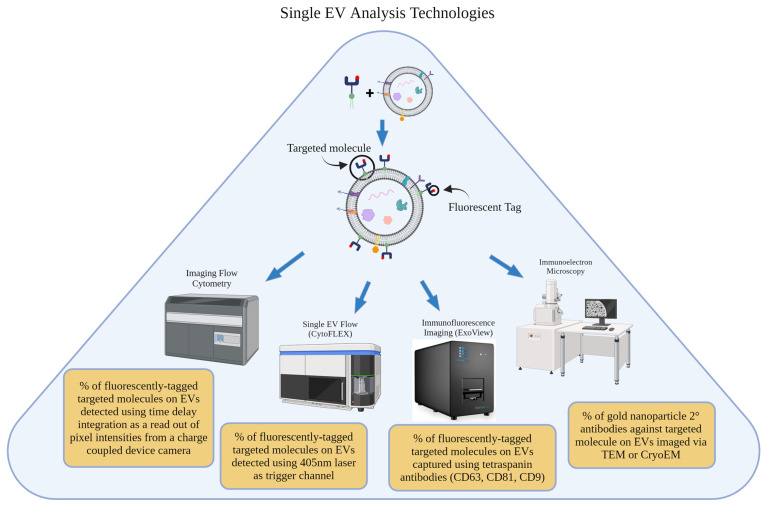
Overview of single EV analysis technologies used to quantify EV conjugation efficiency and the fundamental principle behind each technique’s calculation.

Single-nanovesicle analysis techniques focus on analyzing individual EVs within a sample [53]. These techniques provide the opportunity to calculate the percentage of EVs within one sample that are conjugated with a molecule of interest (Figure 2). Although still new to the field, more studies are adopting the use of these technologies.

### 4.1. Single EV Flow Cytometry

Flow cytometry has previously been used to analyze and measure the characteristics of individual cells in a sample. Flow cytometer instruments suspend cells in a fluid medium and pass them through a laser beam. As the cells pass through the laser beam, they scatter light in different directions and emit fluorescence based on their specific characteristics, including cell size, complexity, and expression of specific markers [58]. Recently, flow cytometers have been adapted and optimized to run nanoparticles such as EVs [59,60,61,62,63,64]. For instance, CytoFLEX flow cytometers can be calibrated such that a 405 nm laser acts as a trigger channel to discriminate EVs from noise. Other instruments, such as NanoFCM [65] and ImageStream^x^ MkII—or Imaging flow cytometry [60] (ISX, EMD Millipore, Seattle, WA, USA)—have also been adapted for the detection and characterization of EVs/nanoparticles. Pham et al. used the CytoFLEX flow cytometer (Beckman Coulter, Brea, CA, USA) to calculate conjugation efficiency on red blood cell-derived EVs modified with biotinylated TR5 peptide via OaAEP1 ligase-aided conjugation. The authors reported that 77.2% of their RBCEVs were TR5-ligated compared to uncoated RBCEVs (at 4.07% detected conjugation) [30]. This group continued on to engineer RBCEVs with two other biotinylated peptides—B-TL5 and B-T140—and optimize the ligation protocol to reach roughly 95% and 99% conjugation efficiencies, respectively, compared to unligated EVs [27]. Another study used nFCM technology—a flow cytometer commercialized by the authors who co-founded NanoFCM Inc.—(Xiamen, China) to determine the conjugation efficiency of both an Alexa Fluor 555 molecule (Thermo Fisher Scientific, Waltham, MA, USA) alone as well as an Alexa Fluor 555-labeled transferrin ligand onto food-derived EVs (FDEVs). Via the use of this technology—which uses a smaller flow channel to reduce background signal and a continuous-wave solid-state laser to excite single EVs for detection using two single-photon counting avalanche photodiodes—the authors reported that 85% of FDEVs were conjugated with the Alexa Fluor 555 molecule, and 74% of FDEVs were conjugated successfully with transferrin. In this study, the researchers were also able to identify a plateau of conjugation optimization via the increase in maleimide-modified transferrin until a surface crowding effect was observed [37]. Imaging flow cytometry (IFC) has also been utilized as a single-nanoparticle analysis technique employing Time Delay Integration as a read-out of pixel intensities from a charge-coupled device where every object that flows through the system is imaged, negating the need for separate data acquisition triggers [60]. In a study where CFSE-labeled PD-1-conjugated EVs were fused with a cargo-loaded Cy5-liposome, IFC was used to detect a 37.0% fusion efficiency when nanoparticles were co-incubated under acidic conditions (pH 4.5) compared to only 3.78% fusion efficiency under neutral conditions (pH 7.5). This was an important implication in the stability of hybrid EVs during cell uptake since the pH in late endosomes and lysosomes is acidic (pH 4–5), and fusion with acidic organelles is essential for endosomal escape [29].

### 4.2. ExoView/Single Particle Interferometric Reflectance Imaging (SP-IRIS)

Immunofluorescence imaging has emerged as a powerful tool to visualize and confirm biomarker expression in EVs [66]. The ExoView technology offers high-resolution imaging and single vesicle enumeration, thus improving the accuracy of EV subpopulation analysis. This technology uses similar principles to a sandwich ELISA. An ExoView chip has spatially distinct spots that are typically pre-coated with normal EV tetraspanin capture antibodies CD9, CD63, and CD81. EVs are then incubated on the chip to be immobilized. Then, the captured EVs are probed for protein expression and marker colocalization using fluorescent detection antibodies against biomarkers of interest. Many studies have employed this technology to investigate the percentage of EV markers including tetraspanins or essential therapeutic receptors/growth factors. These studies have been vital in identifying EV markers for cancer diagnostics, elucidating the function of biomarkers, and revealing key insights into EV biogenesis pathways [66,67,68,69]. However, few studies in the literature have taken advantage of this technology’s potential for use in EV surface conjugation analysis. In a study where placental-derived mesenchymal stromal cell EVs (PMSC-EVs) were conjugated with a collagen-binding peptide SILY via click chemistry and amine-aided crosslinking, researchers used a TAMRA (552 nm)-labeled SILY peptide for detection using the ExoView R100 equipment. The use of a fluorescently tagged anti-CD63 antibody allowed for the total count of EV particles, while the number of TAMRA+EVs served as the precise number of EVs labeled with this SILY peptide. With these measurements, researchers quantified the efficiency of the EV/SILY conjugation protocol to be roughly 70% SILY+EVs. A control sample of EVs incubated with TAMRA-SILY without the presence of the dibenzocyclooctynesulfo-N-hydroxy-succinimidyl ester (DBCO-sulfo-NHS) linker demonstrated a loss of conjugation efficiency proving that calculated efficiencies were true [28]. This technology has been verified via this study to confirm the degree of successful surface modification of targeting molecules onto EVs.

### 4.3. Immunoelectron Microscopy/Immunogold Labeling

Immunoelectron microscopy has been employed to observe successful surface modification of nanoparticles on a single vesicle level [70]. In most instances, particles are incubated on electron microscopy grids. Samples’ conjugated molecules are then labeled with primary antibodies against the exact peptide, HA tags, or any other epitope modified on conjugated ligands. Finally, secondary antibodies conjugated with gold nanoparticles bind the primary antibodies, and grids are imaged, most commonly using transmission electron microscopy or cryogenic electron microscopy to observe the presence of gold nanoparticles bound to nanoparticles. Most studies show the presence of gold nanoparticles to show successful surface conjugation, but in all instances, less than 100% of the nanoparticles imaged are successfully conjugated. Several reasons could explain this occurrence. Some studies vary parameters in the conjugation methods and show a correlation with conjugation efficiency [43]. Others suggest that protein dissociation from membranes could occur during electron microscopy processing [40]. It is also important to note that across literature that employed immunogold labeling, there is no standardized minimum of nanoparticles to be counted for adequate conjugation analysis. This, along with differences in nanoparticle surface modification methods, could lead to inconsistent results between reported surface conjugation efficiencies. Studies have used biotinylated aptamers incubated with EVs, followed by the use of streptavidin-conjugated gold nanoparticles to show successful surface conjugation [47]. Other studies have conjugated EVs with fluorescent markers (e.g., GFP or tdTomato) and have used anti-GFP or anti-tdTomato secondary antibodies conjugated with gold nanoparticles to visualize successful EV conjugation [71]. Although TEM is limited in its throughput, visualization of individual gold nanoparticles on nanoparticle surfaces can give researchers helpful insight into where conjugated molecules of interest are located within vesicle membranes. Another study used anti-HA antibodies followed by anti-IgG conjugated with gold particles to confirm the presence of transfected GE11 and EGF onto EVs. They estimated that 15.3% and 21.2% of the EV samples were modified with GE11 and EGF, respectively [39]. Similarly, another study reported a similar 15–25% of EVs that displayed at least 1 EGa1 nanobody, indicated by at least one gold nanoparticle observed on EV surfaces in TEM images [25]. In contrast, another study estimated only 7–14% of their conjugated EVs contained at least one EGa1 nanobody. Researchers also reported that an average of 0.4–4 nanobodies were present on each EV [43].

The two previously reported calculations [43] represent two major schools of thought when it comes to reporting nanoparticle conjugation analysis. Reporting the percentage of total EVs labeled with one conjugated molecule can give insight into the degree of nanoparticle conjugation required to see the desired effects of a nanotherapeutic in vitro and in vivo. However, an assumption made with this reported percentage is that nanoparticles only need one targeting molecule to be conjugated on their surface to improve their individual targeting ability. When reporting the average number of molecules conjugated onto each nanoparticle, researchers can understand how many targeting molecules are generally present in one nanotherapeutic dose. An assumption made with this reported average is that most, if not all, of the nanoparticles in the sample are at least labeled with some number of targeting molecules. As has been made clear—especially in immunogold labeling TEM images—no conjugated sample has every particle modified with the molecule of interest, and therefore the previous assumption is unreliable.

## 5. Improved Targeting/Uptake/Biodistribution

Once the surface modification of extracellular vesicles is complete, a critical assessment step is to evaluate their targeted delivery efficacy. Some groups have immobilized a molecule of interest onto slides and used fluorescence microscopy to observe conjugated nanoparticles incubated and immobilized onto the coated slides [72]. Some of these studies include observed collagen-binding abilities of SILY-EVs [28], Nucleolin-targeting aptamer conjugated ENVs via immobilization via complementary DNA probes [26], and liposomes’ HER2 targeting ability using anti-HER2 Fab conjugation using surface plasmon resonance [36]. One study even quantitatively analyzed fluorescent images of immobilized CpG DNA-conjugated EVs to report an average of 287 CpG DNA molecules present per EV. These data were calculated based on measurements of EV concentration and protein content as well as the fluorescent intensity of CpG DNA molecules [48].

Studies have also followed up by analyzing cellular uptake or biodistribution in target cells or animal models, respectively. Flow cytometry has been used effectively to confirm the interaction of modified vesicles with their intended target cells in vitro, providing quantitative data on the level of targeting molecule conjugation and the efficiency of the modification process [36,40,41,44,72]. Others use ex vivo [33] or in vivo [35,37,41,72] approaches to confirm proper or improved biodistribution among tissue or organs of interest. Although these methods may not serve as an exact calculation of conjugation efficiency, researchers are able to better understand how the degree of conjugation affects the targeting ability and function of surface-conjugated molecules. This discussion of how conjugation efficiency correlates with improved nanoparticle targeting is essential to this growing field—especially as research groups plan for the use of their therapeutics in clinical trials and eventual use in the clinic.

## 6. How Conjugation Efficiency Calculations Differ Based on Confirmation Method

Because so many different technologies and assays have been devoted to calculating conjugation efficiency and confirming successful EV surface modification, there are several other factors of note that can contribute to differences in reported conjugation results. It is worth noting that each technique has limitations and differences in such variables as vesicle heterogeneity/subpopulation sampling, definition of conjugation efficiency, and equipment’s detection thresholds can drastically affect understanding of EV surface modification [73]. In this section, we will discuss these factors and how they may change conjugation calculations.

### 6.1. Sampling of Different Total Populations

A common fraction used in conjugation efficiency calculations is shown below.

(1) $$\frac{\#\;Nanoparticles\;Positive\;for\;Conjugated\;Molecule}{Total\;\#\;of\;Nanoparticles}$$

In many cases, the method of nanoparticle formation, EV isolation method, or cell-culture conditions chosen in a study can affect the denominator of this fraction [74,75]. Furthermore, if the technology used measures the total # of nanoparticles via methods that favor certain EV subpopulations (e.g., based on biomarker expression), this fraction may also change. For instance, certain studies have used CD63-coated beads for bead-based flow cytometry. The use of this tetraspanin can preferentially select a CD63+EV population, causing downstream changes to the calculated conjugation efficiency [35]. Additionally, label-free nanoparticle detection technology has been shown to be less sensitive than fluorescence-based technologies. Therefore, technology such as ExoView may return a higher total nanoparticle count despite using capture antibodies that would normally imply capturing only a subset of EVs in a sample that expresses such antigens [76].

### 6.2. Detection Threshold of Technology

Another factor that should be considered in the calculation of conjugation efficiency is the various technological detection thresholds with regard to resolving nanoparticle size. EVs have been reported to span many size ranges (depending on the biogenesis pathway), and technologies used to characterize nanoparticles may not be able to resolve down to the smallest EV sizes, also known as exosomes (30–150 nm) [75]. Each technology mentioned in this review has a biological particle size detection range (diameter), which can lead to inconsistencies in the nanoparticle sizes analyzed for conjugation efficiency. A higher detection threshold limits the total number of nanoparticles potentially counted in samples and can wrongfully detect a higher percentage of conjugated EVs. Table 2 details previously reported detection thresholds with regard to resolving nanoparticle diameter (nm) [76,77,78,79].

## 7. Future Perspectives and Conclusions

EVs and other nanotherapeutics have great potential to become effective cell-free therapies. Their use has been explored among numerous applications including cancer, osteoporosis, neurodegenerative disease, and immunotherapy. Currently, many studies have modified the surface of nanoparticles to improve the efficacy and efficiency of drug delivery and targeting in vitro and in vivo. However, there are still gaps in current research to standardize practices for confirming conjugation efficiency. To calculate this percentage or number of molecules per particle, techniques will have to explore the use of single-nanoparticle analysis technologies to resolve conjugation at the nanoscale level. Technologies such as single EV flow cytometry and ExoView (SP-IRIS) are pioneers in this field by using fluorescently tagged ligands and single-nanovesicle detectors to more accurately count percentages of single nanoparticles conjugated with molecules of interest. Immunoelectron microscopy—with the use of gold nanoparticles to label conjugated molecules—is also a way to see individual particles’ status of conjugation or lack thereof. However, immunogold labeling can be limited in its rigor due to the smaller sample size compared to single EV flow cytometry and ExoView. Super-resolution imaging is a new potential technology that could be adapted for conjugation efficiency calculation, although it has mostly been utilized to study EV biomarker expression [69] and fluorescent GFP-loaded cargo [80] thus far. This technology would enable the visualization of individual vesicles down to a scale that could not only resolve how many particles in a sample are positive for fluorescently labeled ligands but to determine how many ligands are present on a single nanoparticle. This method also has similar sample sizes to single EV flow and ExoView ranging from hundreds to thousands of particles able to be analyzed within one sample. Aside from confirming and calculating the degree of nanoparticle surface conjugation, these single-nanoparticle analysis technologies have the potential to also be used for confirming the loading of drugs or cargo within nanocarriers. Studies have already begun using ExoView, NanoFCM, and Western blot analysis to confirm the presence of GFP-loaded EVs and, in doing so, calculated both percentages of GFP+EVs as well as the average number of GFP molecules present within each EV [81]. Studies have also used methods mentioned in this review to identify enriched proteins in the EV subpopulation that had been ligated with targeting proteins [30]. Considering the many single-nanovesicular analysis technologies developed, the potential to inform the optimization of conjugation and to correlate the degree of surface conjugation required to see therapeutic effects in vivo is endless. With the continuation of research within this field, targeted nanotherapeutics could overcome the previous challenges of inadequate drug delivery and provide the next steps to develop enhanced and efficient therapy.

## Figures and Tables

**Table 1:** Summary of EV/nanoparticle conjugation confirmation methods and efficiency calculations.

Ref.	Application	EV Source/ Nanoparticle	Targeting Molecule	Conjugation Method	Conjugation Confirmation Method	Reported Conjugation Efficiency
[38]	Alzheimer’s	Immature dendritic cells (DCs)	RVG; MSP; FLAG	Cell transfection with lamp2b	qPCR; WB	N/A
[30]	Lung Cancer	Red blood cells (RBCs)	EGFR-targeting peptide (ET); TR5	Sortase A & OaAEP1 ligase-aided conjugation	WB (HRP serial dilution); Bead-based FC; Single EV FC	380 ET peptides/EV; 77.2% TR5-ligated EV
[39]	Breast Cancer	HEK293 cells	GE11; EGF	Cell transfection with pDisplay	WB; Bead-based FC; Immunogold labeling + TEM	15.3% (GE11); 21.2% (EGF)
[25]	Tumors/ Cancer	Neuro2A cells	EGa1 (anti-EGFR) nanobodies	Cell transfection, anchored to glycosylphosphatidylinositol (GPI)	WB; Immunogold labeling + TEM	15–25% EGa1
[40]	Tumors/ Cancer	RBCs; Neuro2A cells	EGa1	Direct binding to phospholipid phosphatidylserine (PS) via lactadherin (C1C2)	WB; Immunogold labeling + TEM	Amount of EGa1-C1C2 conjugated to EVs increased in concentration dependent manner
[41]	Cardiovascular disease	Cardiosphere- derived cells	ASSLNIA (muscle homing peptide); CSTSMLKAC (ischemic targeting peptide)	Lipid insertion; Streptavidin-biotin binding	Cell Uptake	N/A
[42]	Melanoma	B16F10 cells	Cy3 fluorophore	Metabolic labeling with azide; Click chemistry	Cy3 absorption using Nanodrop; Bead-based FC	790 nM AHA-Cy3/EVs (1mg/mL); 440 nM ManNAz-Cy3/EVs (1mg/mL)
[43]	Tumors/ Cancer	Neuro2A cells	EGFR ligand (EGa1)	Micelle formation by DSPE-PEG-maleimide + SH-ligand; Fusion	WB; Immunogold labeling + TEM	7–14% EGa1; 0.4–4 ligands/EV
[44]	Cerebral Ischemia	Bone marrow mesenchymal stromal cells (BMSCs)	αvβ3-targeting peptide c(RGDyK)	Click chemistry; Amine crosslinking	Standard curve determined by FITC-labeled c(RGDyK)	263 peptides/EV
[33]	Immunotherapy	Bovine serum	Mannose	Amine crosslinking (NHS-PEG-biotin); Lipid Insertion (DSPE-PEG-biotin or DSPE-PEG-mannose)	Gel electrophoresis; Biotin quantification kit	40% NHS-PEG-bio on EVs; 70% DSPE-PEG-bio in EVs; ~12-250 nmol/mg EV (DSPE-PEG-biotin); ~40-400 nmol/mg EV (NHS-PEG-biotin) * depending on incubation ratios
[35]	Immunotherapy	THP1; J774A.1 cells	FasL; AS1411 aptamer; Cy5	Cholesterol modification + ssDNA tether	Bead-based FC (CD63 antibody); Fluorescence microscopy	1800–6900 ssDNA tethers/EV * depending on concentration of Chol-DNA
[45]	Abdominal aortic aneurysms	BMSCs	Cathepsin K binding peptide (CKBP)	Click chemistry; Amine crosslinking	Fluorescent plate reader (standard curve)	0.22 μg CKBP per 10 μg EV
[36]	HER2-overexpressing cancer	Liposomes	Anti-HER2 Fab’ fragments	Lipid Insertion; Maleimide-thiol crosslinking to Fab’ fragments	Dye binding (protein concentration) assay	100–120 Fab′ fragments/liposome
[28]	Muscular damage, inflammation, cirrhosis	Placental-derived MSCs (PMSCs)	SILY peptide	Click chemistry; Amine crosslinking chemistry	Fluorescence microscopy; ExoView	70% SILY peptide
[27]	Myeloid leukemia	RBCs	B-TL5; B-T140	OaAEP1 ligase-aided conjugation; Streptavidin-biotin binding	WB (HRP serial dilution); Single EV FC; Competitive ELISA	95% B-TL5; 99% B-T140; 351 B-TL5/EV; 1000-1402 B-T140/EV
[37]	Tumors/ Cancer	Food-derived (milk & plant cells)	Alexa Fluor 555; Transferrin	Disulfide reduction via mild reducing agent TCEP; Thiol-maleimide conjugation	Fluorescence spectrometry + NTA; ELISA; NanoFCM	Max of 1965/mEV; 85% Alexa Fluor 555; 74% Transferrin
[46]	Breast Cancer	HEK293FT cells	Tumor-homing peptides (THPs) – PDL1; uPAR; EGFR	Lipid Insertion; Amine-crosslinking chemistry	N/A	N/A
[47]	Liver Cancer	HepG2 cells	Nanoassembly (NA)-(HepG2 EV-binding aptamer LZH8 & M1/M2 monomers)	HepG2 EV-binding aptamer LZH8; DNA Hybridization Chain Reaction (HCR)	Bead-based FC; Immunogold labeling + TEM	N/A
[29]	Cancer	Sf9 cells	Programmed cell death 1 (PD-1) protein; Baculoviral fusogenic glycoprotein gp64	Cell transfection with plasmid transformed into recombinant baculoviruses	WB; Imaging FC (IFC)	37% fusion efficiency with PD-1 EVs and cargo-loaded liposomes
[31]	Breast Cancer	PLGA–PEG nanoparticles (NP)	Monoclonal antibody TRAZ (antibody fragment)	Disulfide-selective pyridazinedione linkers; Click chemistry	BCA (standard curve); Surface plasmon resonance (SPR)	193.1 TRAZ F(ab) pmoles/mg PLGA-PEG NPs; 18.4% modified TRAZ F(ab)
[32]	Cancer	Polyion complex (PIC) micelles	Anti-EphA2 (antibody fragment)	Maleimide-thiol crosslinking; Click chemistry	UV-vis absorbance	1.5–3.5 Fabs/micelle
[48]	Cancer Immunotherapy	B16BL6 cells	CpG DNA	Streptavidin (SAV) cell transfection; Streptavidin-biotin binding	Fluorescence microscopy	287 CpG DNA molecules/EV
[34]	Colorectal Cancer	BM-MSCs	MUC1 aptamer	EDC/NHS chemistry	Nanodrop; Gel retardation assay (gel electrophoresis)	44.78 μg MUC1 aptamer/100 μg EV
[24]	Osteoporosis	RBCs	TBP	CD63 receptor binding through CP05 peptide	Bead-based FC	N/A
[26]	Cancer	Immature mouse dendritic cells	Nucleolin-targeting aptamer AS1411	Cholesterol anchor on cells; Extrusion to form exosome-mimetic extracellular nanovesicles (ENVs)	Fluorescence microscopy & Dot blot using Cy5-labeled complementary DNA probe of AS1411	15–25% on cells prior to ENV extrusion * depending on chol-PEG_2000_ concentration
[49]	General targeting	4T1 cells	Fluor 545	EDC/NHS chemistry; Click chemistry	HPLC/UV–vis absorbance	1.5 alkynes available to react to azide-fluor 545/EV (150kDa protein)
[50]	Osteoporosis and fracture	BMSCs	BMSC-targeting DNA aptamer	Amine-based Schiff base reaction	Bead-based FC; Cell Uptake	N/A

**Table 2:** Technologies used to confirm EV/nanoparticle surface conjugation and detection thresholds.

Technology	Detection Thresholds (nm)
SP-IRIS (ExoView)	50 nm
Single Flow Cytometry (CytoFLEX)	70 nm
NanoFCM	40 nm
Conventional Flow Cytometers (Bead-based FC)	200 nm
TEM	1 nm
NTA	70 nm
MRPS	50 nm

## Data Availability

An extensive electronic literature search was conducted on the following databases, PubMed, GoogleScholar, and ResearchRabbit, using the following terms: conjugation efficiency, extracellular vesicle, exosome, surface modification, functionalization, and quantification. The search was performed from November 2023 to March 2024. A reviewer (L.G.-H.) screened the titles and methods section of eligible articles that discussed the quantification of EV/nanoparticle conjugation. The papers were downloaded via the Mendeley application and categorized based on the quantification method. It is important to note that the nomenclature of extracellular vesicles has changed over time, which may limit the comprehensiveness of this study. A standard term for EV conjugation efficiency or quantified surface functionalization has also not been well established in the literature, so the electronic literature search may have missed studies that refer to this calculation differently. At this time, all efforts have been employed to describe the diversity of techniques used for surface modification efficiency calculations.
